# Detection of carbapenem-resistant Enterobacterales from nursing home wastewater effluent from September 2021 to November 2021

**DOI:** 10.1017/ash.2023.235

**Published:** 2023-09-29

**Authors:** Susanna Lenz, Lauren Franco, Angela Coulliette-Salmond

## Abstract

**Background:** Surveillance and early detection of antibiotic resistance genes and multidrug-resistant organisms (MDROs), such as carbapenem-resistant Enterobacterales (CRE), are important to quell outbreaks early, as antibiotic resistance continues to be an increasing threat. Wastewater surveillance in general has gained attention in the United States, but scientific evidence demonstrating the feasibility to assess antibiotic resistance genes and MDROs is limited. In this study, wastewater effluent was used to screen a nursing home facility, which housed a population at increased risk for colonization with MDROs, for the presence of β-lactam–resistant genes.

**Methods:** Wastewater effluent samples (24-hour composite, n = 19; grab samples, n = 6) collected from a skilled nursing home facility from September 2021 to November 2021 in DeKalb County, Georgia, were screened for carbapenem-resistant and extended-spectrum β-lactam (ESBL)–resistant Enterobacterales using 2 selective chromogenic media: mSuperCARBA (mSC) for selection of CRE and CHROMagar ESBL for selection of gram-negative bacteria producing extended-spectrum β-lactamases. Colilert-18 (IDEXX) was applied to detect and quantify total coliforms and *Escherichia coli*, serving as an enrichment approach for potential gram-negative organisms (Enterobacterales) containing antibiotic resistance genes. *E. coli*–positive Colilert-18 (IDEXX) samples (n = 24) had a composite of 1.0 mL total from 5 positive wells or 20% per plate collected and stored at −80°C in 25% glycerol. The *E. coli*–positive Colilert-18 samples were later thawed and plated on mSC and CHROMagar ESBL, where a random subset of all the colonies (ie, mixture of typical and atypical colonies; n = 28) were selected for matrix-assisted laser desorption/ionization time-of-flight (MALDI-TOF) to confirm identification. Additionally, a non-enrichment approach, filtered wastewater samples (10 mL, n = 23) were frozen (−80°C) until DNA extraction, followed by multiplex real-time PCR for the *bla*KPC, *bla*NDM, *bla*VIM, and *bla*OXA-48–like carbapenemase genes. **Results:** Among 24 *E. coli*–positive Colilert-18 samples, 16 (67%) of 24 contained carbapenem-resistant *Klebsiella, Enterobacter, or Citrobacter* (KEC), 88% contained ESBL-resistant KEC (21 of 24), 4% (1 of 24) contained carbapenem-resistant *E. coli*, and 67% contained ESBL-resistant *E.coli* (16 of 24). In the 28 colonies picked from mSC or ESBL, 10 different genera were confirmed using MALDI-TOF: *Aeromonas*, *Citrobacter*, *Enterobacter*, *Escherichia*, *Klebsiella*, *Providencia*, and *Raoultella*. Of 23 filtered samples, 18 (78%) were positive for the *bla*KPC gene, whereas all samples were negative for *bla*NDM, *bla*VIM, and *bla*OXA-48–like genes. In this nursing home, these findings suggest a concerning frequency of bacteria resistant to last-line antibiotics. Wastewater surveillance can potentially serve as an approach to identify antibiotic resistance and track its presence over time.

**Disclosure:** None

